# Integrative and conjugative elements carrying high-level gentamicin resistance genes in Streptococcus dysgalactiae subsp. equisimilis from horses

**DOI:** 10.1099/mgen.0.001722

**Published:** 2026-05-27

**Authors:** Tony Rochegüe, Sandrine Hughes, Estelle Saras, Benjamin Gillet, Antoine Drapeau, Philippe Glaser, Jean-Yves Madec, Marisa Haenni, Agnese Lupo

**Affiliations:** 1 ANSES - Université de Lyon, Unité Antibiorésistance et Virulence Bactériennes, Lyon, France; 2 Institut de Génomique Fonctionnelle de Lyon (IGFL), CNRS UMR 5242, Ecole Normale Supérieure de Lyon, Université Claude Bernard Lyon 1, Lyon, France; 3 Ecology and Evolution of Antibiotic Resistance Unit, Institut Pasteur, Université Paris-Cité, CNRS UMR6047, Paris, France

**Keywords:** *aac*(6′)-*Im*, *aph*(2″)-*Ib*, ICE*Er0106*, *Streptococcus dysgalactiae* subsp. *equisimilis *(*SDSE*), Tn*5252*, Tn*GBS-2*

## Abstract

In streptococci, the acquisition of high-level gentamicin resistance (HLGR) abolishes the synergy with beta-lactams, constituting a medical concern for humans and animals. This synergy between gentamicin and beta-lactams is useful to treat severe infections like endocarditis, among others. HLGR has been characterized mostly in roup B Streptococci mediated by the bi-functional gene *aac*(6)-*aph*(2).

HLGR–*Streptococcus dysgalactiae* subsp. *equisimilis* (*SDSE*) (*n*=27) were isolated from the uterus of mares during a routine screening before *in vitro* fecundation. One HLGR–*SDSE* was isolated from a lymph node of a mare. The mares resided in six different studs located in four departments of France. HLGR*–SDSE* belonged to three sequence types (STs): ST196 (*n*=26), ST223 (*n*=1) and a single locus variant (SLV) of ST200 (*n*=1). For the first time, HLGR was found mediated by the two genes *aph*(2″)-*Ib* and *aac*(6’)-*Iam* located on a class 5 integrative mobilizable element (IME), here named HLGR_IME. In HLGR–*SDSE* assigned to ST196 and ST223, the HLGR_IME was inserted in a Tn*5252*-type transposon. In the isolate assigned to an ST200-SLV, the HLGR_IME was inserted in an ICE*Er0106*-type element. The Tn*5252*-type transposon was able to transfer *in vitro* from HLGR*–SDSE* to an erythromycin-resistant *Streptococcus agalactiae* strain. The transferability of HLGR from *SDSE* to human pathogens, such as *S. agalactiae*, is a major concern for health. The phylogenetic analysis displayed that *SDSE* from horses constituted a distinct phylogenetic clade from *SDSE* isolated from humans or other animals. Furthermore, we demonstrated that HLGR–*SDSE* belonging to ST196 were able to disseminate among mares within a single stud during 2 years. This clone has also been reported in the USA.

Hygiene measures could prevent further dissemination of HLGR streptococci in veterinary settings, preserving horses’ health, and the transfer of HLGR to pathogens important in veterinary and human settings.

**Impact Statement**

This study is important because it describes for the first time the characterization of the genetic basis underlying high-level gentamicin resistance (HLGR) in *Streptococcus dysgalactiae* subsp. *equisimilis* (*SDSE*) colonizing the uterus of mares. HLGR is of concern for the health of humans and animals, because it impedes the implementation of antibiotic therapies necessary to treat severe infections. Before this study, HLGR was attributed exclusively to the *aac* (6)-*aph*(2)-bi-functional gene. We unveiled that HLGR in *SDSE* from horses was conferred by *aph*(2″)-*Ib* and *aac*(6′)-*Im* genes. In addition, we characterized the genetic element carrying the *aph*(2″)-*Ib* and *aac*(6′)-*Im* genes, which was a class 5 Integrative and Mobilizable Element (IME) in turn inserted in a Tn*5252*-type transposon. This latter element was able to transfer to *Streptococcus agalactiae*, a species that is pathogenic for humans, to which it conferred HLGR. In addition, we unveiled that an HLGR–*SDSE* ST196 clone was able to disseminate among mares within a single stud during 2 years. Our study highlights the importance of monitoring HLGR, also in veterinary settings. Hygiene practices for horse breeding and antibiotic usage need attention and improvement to avoid further spread of this resistance mechanism. Hygiene and antibiotic practices in breeding horses require attention to minimize the risk of disseminating this resistance mechanism.

## Data Summary

The newly sequenced genomes have been submitted to the National Center for Biotechnology Information (NCBI) and their BioProject number is PRJNA1242720. The publicly available *Streptococcus dysgalactiae* subsp. *equisimilis* genomes from animals and humans used in this study are listed in Table S1. All the supplementary tables are available in the supplementary material file.

## Introduction

Aminoglycosides need an active electron transport chain to enter the bacterial cell and reach their target, the ribosome. The electron transport chain is not fully active in anaerobic facultative bacteria like streptococci [1,2]. For this reason, streptococcal species have a natural decreased susceptibility to aminoglycosides, which nonetheless does not impede a synergy between this antibiotic class and beta-lactams. This synergy is exploited to cure infective endocarditis in humans [3]. The combination of beta-lactams and aminoglycosides is also used in veterinary settings to treat different conditions, such as mastitis, skin and soft tissue infections, pneumonia and urinary tract infections [4].

In streptococci, the acquisition of exogenous genes conferring high-level gentamicin resistance (HLGR) can occur, abolishing the synergy with beta-lactams and constituting a medical concern in both human and veterinary medicine [5]. So far, HLGR in streptococci has been attributed to the bi-functional enzyme AAC(6′)-APH(2) encoded by the *aac* [6]-*aph*(2) gene [6]. This bi-functional gene is located on a Tn*4001* transposon, originally characterized in *Staphylococcus aureus*, integrated in the chromosome [7]. In rare cases, the bi-functional gene has been reported on plasmids [5]. Among streptococcal species, HLGR has been mostly documented from *Streptococcus agalactiae*. Reports from non-*agalactiae* species are sporadic and consist of a clonal group of *Streptococcus pasteurianus* isolates from China in the early 2000s [8] and a *Streptococcus dysgalactiae*, isolated in 1995 in France [9]. All these HLGR streptococci have been found in humans.

*Streptococcus dysgalactiae* subsp. *equisimilis* (*SDSE*) is responsible for diverse infections in humans. Many of them are invasive, like endocarditis [10] or associated with toxic shock syndrome [11], and mainly concern elderly or patients with immunocompromised responses [12]. Furthermore, *SDSE* has been associated with sequelae like post-streptococcal glomerulonephritis or reactive arthritis [13], similarly to infections caused by *Streptococcus pyogenes* [14]. *SDSE* is also capable of colonizing [15] and causing diseases in animals [16]. Healthy horses seem frequently colonized with *SDSE* on their skin and respiratory tract [15]. *SDSE* can also be responsible for strangle-like disease in horses [17] and for endocarditis [18] and lameness in pigs [19]. More recently, lameness associated with abscesses that led to death has been reported in a greater one-horned rhinoceros [16].

Occurrence of antibiotic resistance is rising in *SDSE* [11,20] in both human [21] and animal isolates, with resistance towards tetracyclines as the most prevalent, followed by macrolides, whereas aminoglycoside resistance remains rare [22]. This study aimed to characterize the genetics underlying HLGR and their mobility in *SDSE* isolates. These *SDSE* isolates were collected from horses through Resapath, the network of diagnostic laboratories participating in the surveillance of antibiotic resistance in bacteria from diseased animals in France. Furthermore, in order to understand the dissemination of HLGR, we analysed the genetic content of *SDSE* genomes available in public repositories and evaluated their phylogenetic relationship with isolates from French horses.

## Methods

### Isolates collection

During 2017–2022, 28 streptococci demonstrating resistance to gentamicin (growth at contact with a 500 µg charged disc) were collected at ANSES-Laboratory Lyon. Twenty-seven isolates were obtained during a routine genital tract screening of 27 mares, and one was isolated from a lymph node of a further mare. Twenty-three out of the 28 mares lived in the same stud (A/61). The five remaining mares were resident in different studs located in four cities of at least three departments of France (B/ND, C/61, D/50, E/61, F/24, Table S1, available in the online Supplementary Material). HLGR phenotype corresponding to gentamicin Minimun inhibitory concentration (MIC) value ≥512 mg l^−1^ [23] was confirmed by E-test^®^ (bioMérieux, France). Susceptibility to 15 additional antibiotics was evaluated by disc diffusion (Veterinary CA-SFM 2023, https://www.sfm-microbiologie.org/) using the *Streptococcus uberis* strain CIP103219 as a control.

### Genome sequencing

DNA extraction and purification were obtained using the NucleoSpin Microbial DNA Mini Kit (Macherey-Nagel, France). Construction and sequencing of libraries for short-read sequencing (Illumina, NovaSeq6000, PE 2×150) were outsourced (Institut du Cerveau et de la Moelle épinière, Paris). Quality and number of short reads were evaluated using FastQC (https://www.bioinformatics.babraham.ac.uk/projects/fastqc). Shovill v.1.0.4 software (https://github.com/tseemann/shovill) with default parameters was used to assemble the short reads. The quality of the assemblies was evaluated using Quast 5.2.0 [24].

In addition, long-read sequencing [Oxford Nanopore Technologies (ONT)] was performed for three isolates (#45604, #48904, #49594). To improve read length, short fragments (<1 kb) were first removed from DNA extracts using Ampure XP beads (Beckman Coulter Life Sciences, US) and applying a 0.36× ratio. Libraries were then constructed from ~1 µg of purified DNA using the 1D Genomic DNA by ligation protocol (SQK-LSK109 with EXP-NBD104). Barcoded libraries were pooled and sequenced with MinION on an R9.4 flowcell (FLO-MIN106) and using Guppy version 2.3.1 (https://nanoporetech.com/software/other/guppy) for basecalling. Only passed reads (>Q7) were considered for analyses.

### Bioinformatic analysis and tools

Phylogenetic relatedness among genomes of isolates of this study (*n*=28) and *SDSE* genomes available from public repositories (*n*=86) was investigated by SNP calling. The origins and details of the 114 genomes used are presented in Table S1, with public data retrieved on the 17th of May 2024 and including RefSeq National center for biotechnology information (NCBI) (*n*=68), SRA NCBI (*n*=17) and PubMLST (*n*=1). Genomes were annotated using Prokka v.1.14.6 [25]. A core-genome (genes encoding for proteins sharing 80% identity and occurring at least in 90% of isolates, *n*=1447) was identified using Roary v.3.13.0, which generated a multiple core-genome alignment [26]. Recombination regions were identified and removed by Gubbins v.2.4.1 [27], and a maximum-likelihood phylogeny was constructed using the RAxML tool [28]. A SNP distance matrix was constructed using snp-dists v.0.7.0 (https://github.com/tseemann/snp-dists). The obtained tree was visualized and annotated using iTOL v.7 (https://itol.embl.de/). The identification as *SDSE* of all retrieved genomes (*n*=114) was confirmed using fastani v.1.34 [29]. The level of genome completeness was evaluated using Busco v.5.7.1 [30]. Quality parameters of assembly, completeness and identification of genomes sequenced for this study and for those retrieved from NCBI are reported in Table S1. Sequence type (ST) and the presence of genes conferring antibiotic resistance were determined using StarAMR v.0.10.0 [31]. The assignment of an ST to a cluster was determined by PopPunk v2.7.8 analysis using the core genomes of the 114 *SDSE* analysed in this study [32]. The generated tree was visualized using GrapeTree (https://achtman-lab.github.io/GrapeTree/MSTree_holder.html).

### Analysis of genetic elements harbouring resistance genes and *in vitro* transfer assays

The ability to transfer HLGR *in vitro* from three donor isolates, each belonging to a different ST (#45604, ST196; #48904, ST200-SLV; #49594, ST223), was analysed using the erythromycin*-*resistant *S. agalactiae* [group B *Streptococcus* (*GBS*)] strain #42742, available from the ANSES-Lyon laboratory collection. Mating of *SDSE* donors and the recipient *GBS* strain occurred on brain heart infusion (BHI) agar plates (Oxoid, UK). Transconjugants were selected on BHI agar plates containing erythromycin (100 mg l^−1^) and gentamicin (250 mg l^−1^). Frequency of conjugation was calculated as the ratio of colonies growing on the medium containing erythromycin and gentamicin and the number of donor cells (c.f.u. ml^−1^). Colonies growing on selective plates were sub-cultured and a preliminary identification was achieved by determining the Lancefield group (Streptex, Thermo Scientific, France). The presence of the *aph*(2″)-*Ib* gene conferring HLGR in transconjugants was verified by PCR using primers Aph2-Ib_Fw (5′-CTTGGACGCTGAGATATATGAGCAC-3′) and Aph2-Ib_Rev (5′-GTTTGTAGCAATTCAGAAACACCCTT-‘3).

Genetic elements harbouring genes conferring HLGR were analysed by whole-genome sequencing (WGS) in the three donor isolates and transconjugants for which the presence of *aph*(2″)-*Ib* gene was confirmed by PCR. The three strains were sequenced using both Illumina and ONT (Table S1), which enabled hybrid assembly (Unicycler v.0.4.8) [33] combining the short and the long reads obtained, in order to improve genome completeness. The annotation of the genetic elements was obtained using Bakta (https://bakta.computational.bio/) and curated manually, using the FirmData set for support [34]. Analysis of the genetic elements was carried out using blastn [35] for alignments and Easyfig v.2.5 [36] for plotting.

## Results

### Typing and phylogenetic analysis of *SDSE*

The HLGR phenotype (gentamicin MIC >500 mg l^−1^) was confirmed for the 28 *SDSE* collected from horses. After WGS, they were assigned to three STs (ST196, a single locus variant (SLV) of ST200 and ST223) (Table S1). Twenty-six isolates were assigned to ST196, including 22 isolates collected from mares resident in the same stud (A/61) during the 2017–2019 period. As determined from the core-genome alignment of Roary, including *SDSE* exclusively from French horses, these 22 isolates differed by less than 20 SNPs, and some were indistinguishable (Table S2), suggesting the intra-stud dissemination of a single clone. ST196 *SDSE* collected from farms B/ND, D/50 and F/24 shared high genomic similarity with ST196 *SDSE* from farm A/61, differing by 34–65 SNPs (Table S2). Isolate #53906 from farm E/61 was the most divergent, differing by 57–69 SNPs from the other ST196 *SDSE* from France (Table S2). The remaining two *SDSE* isolates from this study belonged to other STs, including a ST223 (#49594) and a SLV of ST200 (#48904).

**Fig. 1.**
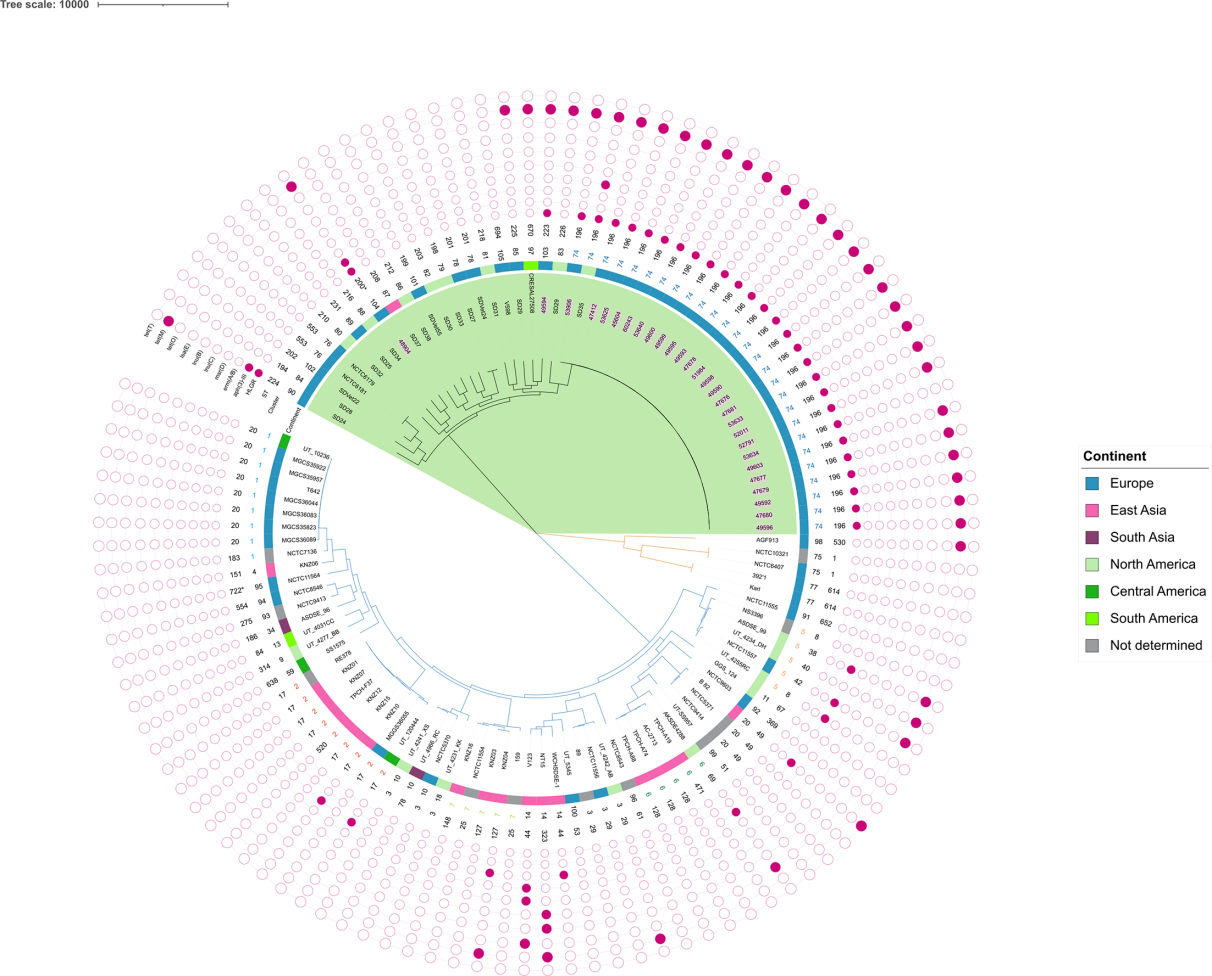
Unrooted phylogenetic tree constructed on SNPs matrix (snp-dists v.0.7.0). Representation and annotation were obtained using iTOL v.7. The node containing genomes from *S. dysgalactiae* sbps. *equisimilis* from equids was highlighted in green. The labels of isolates sequenced for this study are in pink. Orange branches highlighted isolates from various animals, whereas blue branches highlighted isolates from humans. Filled dots of external rings indicate the presence of the resistance genes, with HLGR standing for *aac*(6’)-*Iam* and *aph*(2″)-*Ib* genes. ST: sequence type. Cluster: clustering according to PopPunk core-genome analysis. Clusters containing at least five isolates were coloured. *Single locus variant.

To investigate the genomic proximity of HLGR*–SDSE* from French horses to international *SDSE* from other sources, we conducted a core-genome SNP analysis on the 28 genomes from this study and 86 additional *SDSE* genomes available from public repositories, mostly devoid of HLGR determinants, from equids and other hosts (Fig. 1 and Table S3). The 114 *SDSE* genomes clustered in 3 main nodes, 1 including exclusively isolates from equids (*n*=49), 1 including isolates from various animals (dogs and rhinoceros; *n*=5) and 1 including isolates exclusively from humans (*n*=60) (Table S1). They were assigned to 58 different STs that belonged to several PopPunk clusters (Figs 1, S1 and Table S1).

All *SDSE* from equids, which originated from France (*n*=28), other European countries (*n*=11), North and South America (*n*=21), clustered under the same phylogenetic node (Fig. 1). All SDSE assigned to ST196, including *SDSE* from French mares and SD35 collected from a horse in 2005, in North America, belonged to PopPunk cluster 74 (Fig. 1 and Table S2). Among isolates from non-equid animals, those from rhinoceros were similar to each other at the genomic level and belonged to cluster 77 (Fig. 1). One isolate from a dog and one of unknown origin shared high genomic similarity and belonged to ST1, included in cluster 75. Among *SDSE* from human patients, the largest nodes were made up of isolates belonging to clusters 2 and 1, including nine isolates each. Clusters 5 and 7 included five isolates each (Fig. 1). Genomes included in cluster 2 displayed genomic proximity, despite different geographical origins of the isolates (France, Norway and Texas).

### Antibiotic resistance gene content of *SDSE* genomes

The *tet*(M) gene conferring tetracycline resistance was the most common antibiotic resistance gene found among *SDSE* (*n*=39/114, 34%) and, in particular, in *SDSE* from animals (*n*=32/39, 82%). Other tetracycline-resistance genes, like *tet*(O) (*n*=2/114, 1.8%) and *tet*(T) (*n*=1/114, 0.9%), were sporadic (Fig. 1). The *erm*(B) (*n*=3/114, 2.6%) and *erm*(A) (*n*=6/114, 5.2%) genes were infrequent and detected only in human isolates (Fig. 1 and Table S1). All HLGR–*SDSE* sequenced in the frame of this study harboured the *aph*(2″)-*Ib* gene, located 44 bases upstream from the *aac*(6′)-*Im* gene, and not the bi-functional *aac*(6)-*aph*(2) gene commonly attributed to HLGR in other streptococci [8,37]. These two genes were also present in *SDSE* SD24 and SD35, both collected from horses in Italy and the USA, respectively. These two isolates predated those collected in France, dating back to 2005 (SD35) and 2009 (SD24), respectively (Table S1). While SD35 was assigned to ST196, SD24 belonged to another ST (ST224). Other aminoglycoside resistance genes, like *aph*(3″)-*IIIa*, were sporadic, being only present in the genome SD24 and #48904 of this study.

### Genetic background of the *aph*(2″)-*Ib* and *aac*(6′)-*Im* HLGR genes

**Fig. 2.**
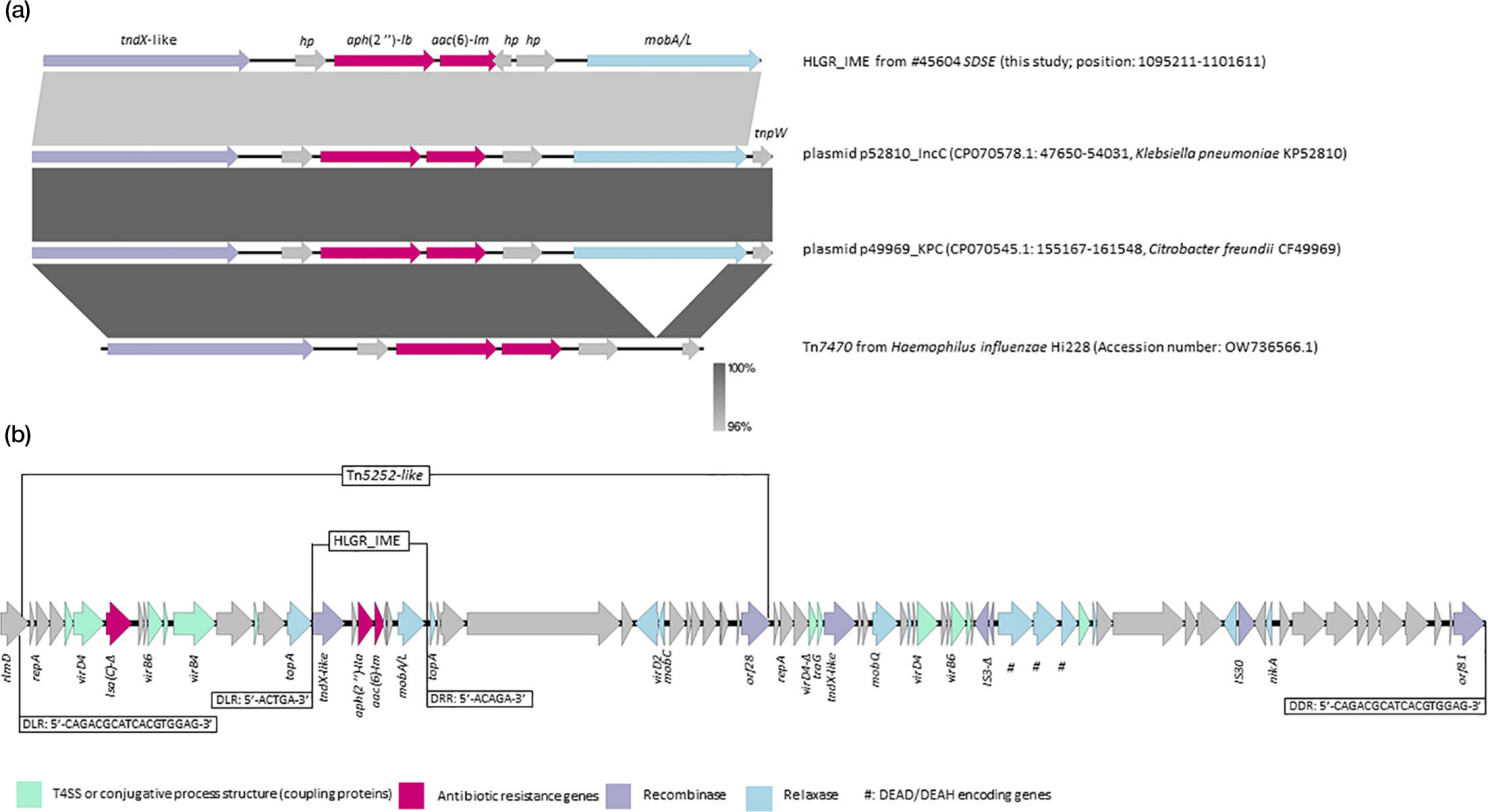
(**a**) Easyfig v.2.2.5 plot of the blastn alignment of the integrative mobilizable element carrying high-level gentamicin resistance genes (HLGR_IME), plasmid fragments from Enterobacterales (p52810_IncC and p49969_KPC), and Tn*7470* from *H. influenzae*. Genes encoding hypothetical proteins were indicated by ‘hp’. (**b**) Plot of DNA coding sequences of Tn*5252*-type carrying the HLGR_IME in #45604 (ST196) *SDSE* and its genetic environment (Easyfig v.2.2.5).

In all HLGR*–SDSE* of this study, as well as in SD35 and SD24 (ST224) from public repositories, the two HLGR genes were located on a 6,398 nt-long fragment, including coding DNA sequences (CDSs) typical of class 5 integrative mobilizable elements (IME) [38]. We named this new IME ‘HLGR_IME’ (Table 1). A blastn search displayed that the HLGR_IME was also located on IncC plasmids (100% coverage and 96% nucleotide identity) co-harbouring carbapenemase-encoding genes in *Enterobacterales* (*Klebsiella pneumoniae* KP52810 (CP070578.1); *Citrobacter freundii* CF49969 (CP070545.1). The HLGR_IME was also similar to the Tn*7470* transposon (accession number: OW736566.1) first characterized in *Haemophilus influenzae* [39] (Fig. 2a).

**Table 1.** Features of genetic elements carrying *aph*(2″)-*Ib* and *aac*(6′)-*Im* mediating high-level gentamicin resistance in *S. dysgalactiae* subsp. *equisimilis* isolates from horses

Isolate		Conjugation component						
	Genetic element	Coupling protein (conserved domain)	Position	Relaxase (conserved domain)	Position	VirB4 position	Recombinase (conserved domain)	Position
#45604-ST196	Tn*5252*-type	CP-IIa (pfam02534)	1081646-1083427	Rel-II (pfam03432)	1113489-1114820	1087334-1089763	Serine integrase (pfam07508)	1119543-1121225
	HLGR_IME	None	–	MobA/L (pfam03389)	1100064-1101611	None	Serine integrase (cd00338)	1095213-1097057
#48904-ST200-SLV	ICE*Er0106*-type							
	Tn*1806*-type	None	–	Rel-II (pfam03432)	1599914-1601248	None	Triplet serine integrase	1589728- 1594554
	Tn*916*-type	CP-I (pfam01580)	1604399-1605784	Rel-I (pfam02486)	1605962-1606996	1608509-1610956	Tyrosine integrase (pfam02920)	1619958-1621175
	HLGR_IME	None	–	MobA/L (pfam03389)	1638379-1639926	None	Serine integrase (cd00338)	
	Tn*GBS2*-type	CP-IIa (pfam02534)	1658201-1659991	None	–	1651548-1653791	DDE integrase (pfam13808)	1664156-1665289

**Fig. 3.**
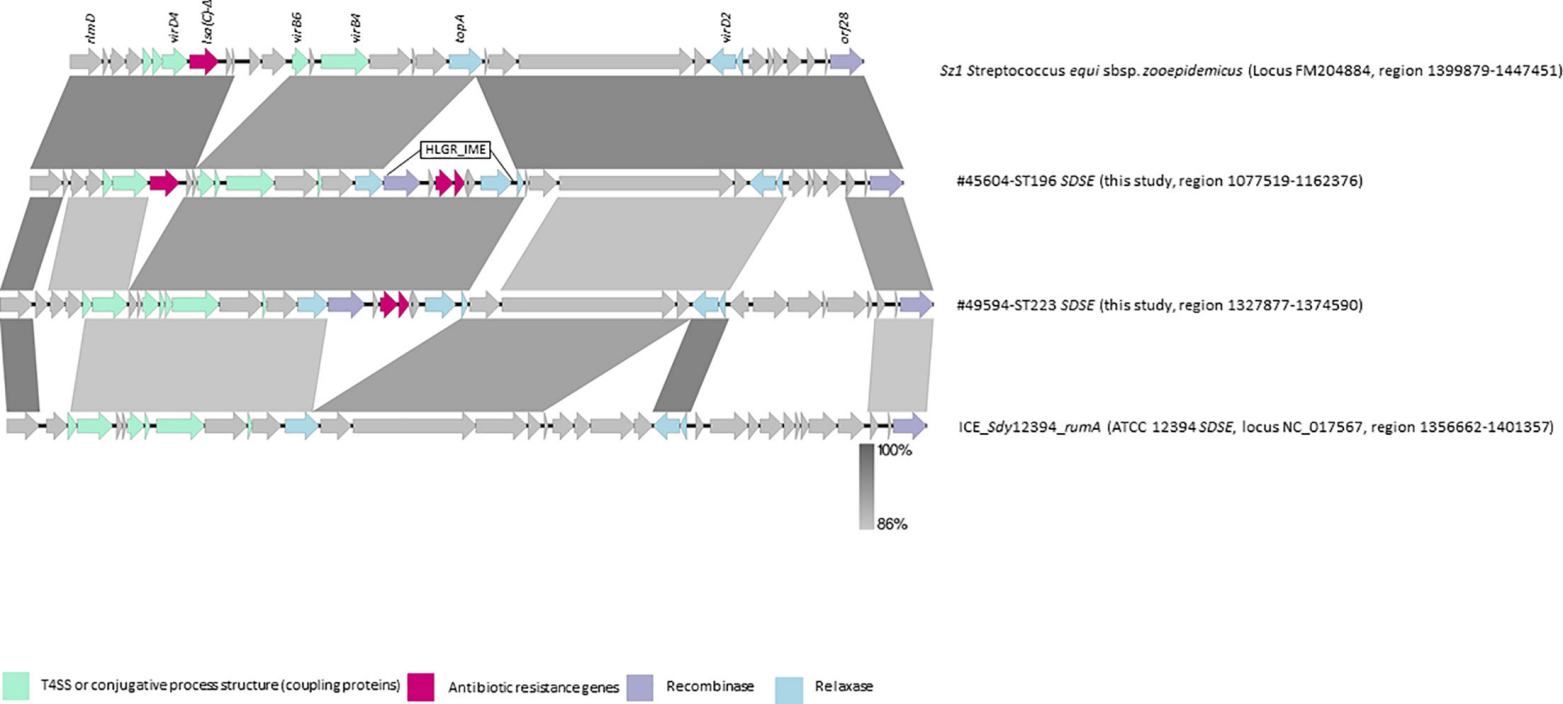
Alignment between Tn*5252*-type from *SDSE* and other similar genetic elements reported in other species (blastn, Easyfig v.2.2.5).

In all ST196 *SDSE* of this study and SD35 (ST196), the HLGR_IME was inserted in a *topA* gene, generating the imperfect duplication of the insertion site (DLR: 5′-ACTGA-3′; DRR: 5′-ACAGA-3′) (Fig. 2b). In turn, the *topA* gene was part of another genetic element, with features of an integrative conjugative element (ICE). This predicted ICE comprised 28 CDS (42,056 nt) including those typical of Tn*5252* ICE superfamily [40], with a recombinase containing a SpoIVCA domain encoded by the here-named ‘*orf28*’ (Fig. 2b). The Tn*5252*-type ICE also carried a *lsa*(C) gene, which confers lincosamides and streptogramins A resistance [41] (Fig. 2b), that was not detected by the StarAMR tool because the gene was devoid of the transcription initiation codon. Indeed, *SDSE* of this study were susceptible to lincosamides and streptogramins A (Table S1). Direct or inverted repeats relative to the integration of the Tn*5252*-type element could not be visualized in the chromosome of #45604 *SDSE* that was fully assembled. The Tn*5252*-type element appeared to be part of a larger element containing a serine recombinase (conserved domain pfam07508), here named ‘*orf81*’ (Fig. 2b). Overall, this 82,567 nt-long composite element was flanked by 18 nt direct repeat sequences (5′-CAGACGCATCACGTGGAG’−3) and was inserted into a *rlmD* gene encoding a 23S uracil(1939)-methyl transferase (Fig. 2b). This element was conserved in all *SDSE* assigned to ST196, including SD35 from the NCBI database, with 100% nucleotide identity and coverage. In the SD35 genome, which is not fully assembled, the Tn*5252*-type sequence matched with two contigs. The Tn*5252*-type element characterized in #45604 (ST196) shared similarities with previously characterized elements like ICE_*Sdy*12394_*rumA* [40], and ICE*Sz1* from *Streptococcus equi* subsp. *zooepidemicus*. A Tn*5252*-type was also present in #49594 *SDSE* (ST223) of the current study, with 85% coverage and 91.6% nucleotide identity (Fig. 3).

**Fig. 4.**
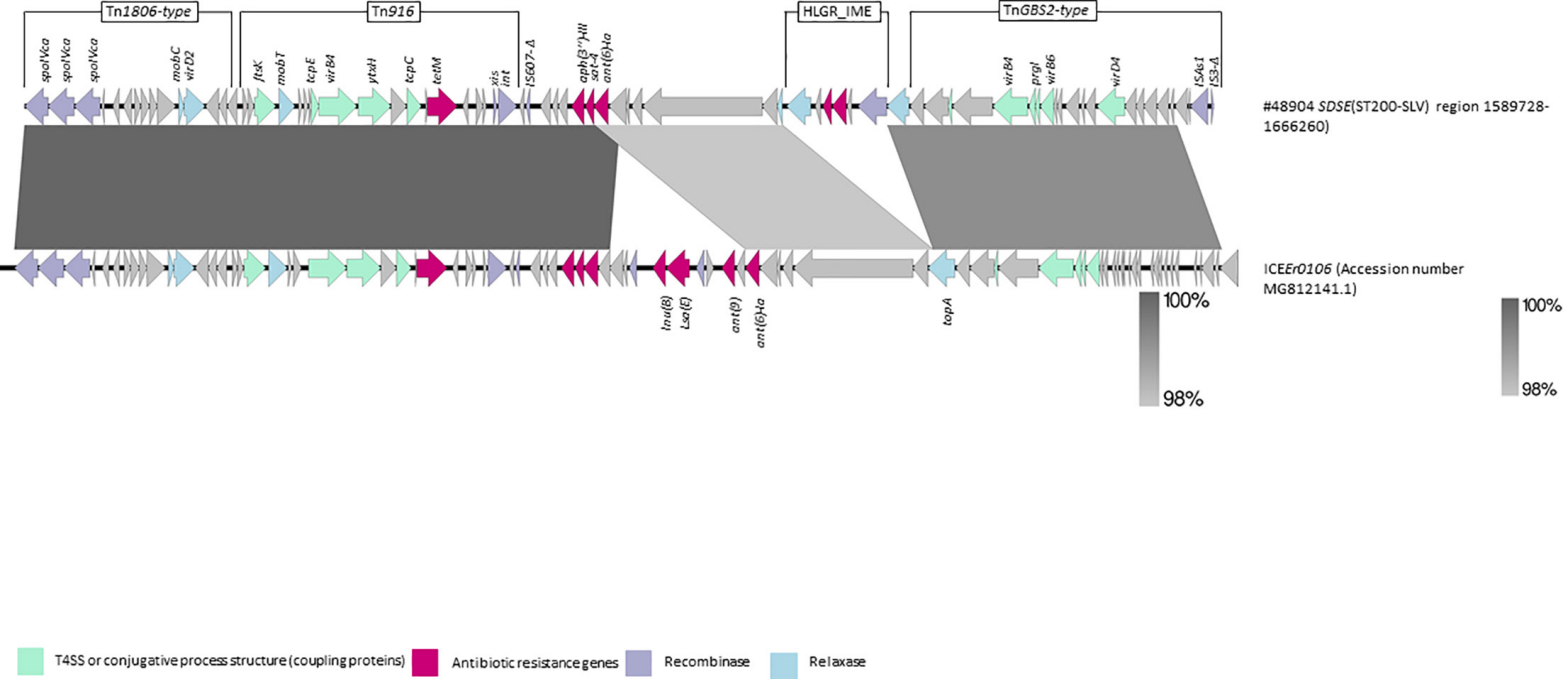
Genetic background of the HLGR_IME in #48904 ST200-SLV *SDSE* and representation of the blastn alignment with ICE*Er0106* from *E. rhusiopathiae* (blastn, Easyfig v.2.2.5).

In isolate #48904 (ST200-SLV), the HLGR_IME was inserted in a different genetic background than Tn*5252*. This novel element shared nucleotide similarity with ICE*Er0106* reported from *Erysipelothrix rhusiopathiae* isolated from a pig with erysipelas (GenBank MG812141.1) (Fig. 4). In turn, this element included CDS typical of the Tn*GBS2*-type transposon [42], of the Tn*916* transposon with the *tet*(M) gene (accession number U09422) and of the Tn*1806* transposon (accession number CP002121.1) [43] (Table 1 and Fig. 4), carrying other resistance genes such as *aph*(3’)-*III*, as well as a *sat-4* gene. Overall, this 76,532 nt mosaic region (position 1,589,728–1,665,658) was inserted between a DNA–methyltransferase-encoding gene and a 2-keto-3-deoxy-d-arabino-heptulosonate-7-phosphate synthase-encoding gene in the chromosome of #48904, but no direct or inverted repeat sequences could be visualized. This mosaic region was present with 99% of nucleotide identity in SD24 (ST224), although the sequence was split into four contigs.

To evaluate the ability of dissemination of HLGR from *SDSE* to other species, we conducted mating experiments using #45604 (ST196), #49594 (ST223) and #48904 (ST200-SLV) as donors and the erythromycin-resistant *GBS* strain #42742 as the recipient. While no transconjugant was obtained from the #49594- and #48904-#42742 mating, the #45604-#42742 mating generated colonies resistant to erythromycin and gentamicin (frequency of conjugation: 15×10^−9^ c.f.u. ml^−1^). These colonies were characterized as *GBS* by Lancefield agglutination and were HLGR (MIC >1,024 mg l^−1^) and erythromycin-resistant (MIC >256 mg l^−1^), suggesting the occurrence of genetic transfer from the HLGR #45604 *SDSE* to the recipient erythromycin resistant #42742 *GBS*. The presence of the *aph*(2″)-*Ib* gene in HLGR–*GBS* transconjugants was verified by PCR. One colony, #63005, positive for the presence of *aph*(2″)-*Ib* gene, was sub-cultured, and WGS was obtained by short-read approach. blastn analysis showed that the Tn*5252*-type element (42,056 nt) was present in the transconjugant, integrated between a Tn*3872* transposon and an *rlmD* gene (Fig. S2). This, therefore, demonstrated that Tn*5252* was able to transfer to the #42742 *GBS* recipient.

## Discussion

In 2024, HLGR has increased among virulent *GBS* in France [44], and, even though sporadically, HLGR has been documented in non-*GBS* streptococci like *S. pasteurianus* and *S. dysgalactiae* [7,8]. HLGR constitutes a health concern, hindering the synergy with beta-lactams to cure life-threatening infections. In *SDSE*, HLGR has been reported from Europe (France and Italy) and the USA, demonstrating a potential global concern. In *SDSE* from horses, HLGR has emerged in multiple phylogenetic sub-nodes, suggesting the occurrence of independent acquisition through mobile elements. While the bi-functional gene *aac*(6)-*aph*(2) mediates HLGR in *GBS*, in *SDSE,* this phenotype was conferred by the *aph*(2″)-*Ib* and *aac*(6′)-*Im* genes. Chow *et al*. have previously reported these genes and their spacing region in *Enterococcus faecium* and *Escherichia coli* [8]. The surrounding genetic environment of *aph*(2″)-*Ib* and *aac*(6′)-*Im* genes in these species remains undetermined. In *SDSE*, the *aph*(2″)-*Ib* and *aac*(6′)-*Im* genes were carried by a HLGR_IME, belonging to class 5 IME and inserted in a *topA* locus predicted to encode a DNA III topoisomerase. A blastn search revealed that this HLGR_IME was found integrated in the *topA* gene also in organisms of the *Enterobacterales* family (*K. pneumoniae* and *C. freundii*) and *H. influenzae,* highlighting that this IME has a large host spectrum and could thus contribute to a wide horizontal dissemination of HLGR. In all these different species, the HLGR_IME was inserted in a *topA* gene, an insertion site unreported so far for class 5 IMEs that have been previously described as inserted in *traG*, *maff2* and *snf2* genes of Tn*5252* superfamily ICE or in chromosomal *rumA* genes [38]. In turn, the HLGR_IME was inserted in different genetic backgrounds in *SDSE* of this study and in genomes retrieved from databases. In ST196 and ST223 *SDSE*, the HLGR_IME was carried on a Tn*5252*-type element, which was inserted in the *rlmD* gene. Transposons of the Tn*5252* superfamily have been crucial for the dissemination of different antibiotic resistance genes in several species of the Bacillota (previously Firmicutes) phylum [40,45]. Differently, in ST200-SLV and ST224, the HLGR_IME was carried by an ICE*Er0106-*type element previously characterized in an *E. rhusiopathiae* isolate. According to a blastn search, ICE*Er0106* includes, in turn, large host spectrum ICEs, like Tn*3872* and *GBS2*.

While no transfer mediated by the ICE*Er0106-*type carrying the HLGR_IME could be observed, the transfer of HLGR through the Tn*5252-*type element from *SDSE* to *GBS* was demonstrated *in vitro*, confirming the role of these ICEs in the dissemination of antibiotic resistance determinants.

Vertical dissemination of HLGR associated with the ST196 clone occurred at an international scale, spanning from the USA to France. Furthermore, in ST196, HLGR can sporadically occur in unrelated isolates, since the isolate from the USA was collected in 2005 and French isolates were collected more than 10 years later. In France, HLGR–*SDSE* ST196 isolates disseminated among mares residing in stud A and persisted for 2 years (2017–2019). Another ST196 HLGR–*SDSE* was recovered in 2020 in another stud located in the same department as stud A. However, this isolate (#53906) diverged from the other ST196 isolates (by 31–37 SNPs), even more than isolate SD35 (18–24 SNPs) collected in 2005 in the USA. On the contrary, the most recent ST196 isolate (#60243) collected in France, in 2022, in another stud and another department than stud A displayed closer genomic similarities with isolates from this latter stud (9–23 SNPs). Most likely, the circulation of the ST196 clone in stud A was favoured by the close proximity of animals, although caring practices, such as vaginal screenings, cannot be excluded from the contribution to the dissemination. The epidemiological connection between #60243 ST196 isolate (stud F) and those from stud A is not evident. In France, *in vitro* fecundation of mares is a frequent practice; thus, the contamination of the semen of a donor stallion could colonize several mares.

*SDSE* isolates from horses, originating from France and from the USA, all belonged to PopPunk cluster 74. This is in contrast with *SDSE* from humans, where isolates causing invasive diseases belonged to different clusters [46]. The core genome of isolates from horses segregated from that of isolates of other hosts. The observation of the current study corroborates previous reports, where a divergence of genomes of *SDSE* isolates found in horses from those isolated from humans and other animals was observed, suggesting the adaptation of a clade of *SDSE* to horses [47,48]. The fact that V598 from a donkey clustered with isolates from horses suggests that host adaptation has probably occurred for the equids family. This hypothesis should be confirmed by adding more genomes from donkeys and zebras. Host-specialization seems to occur from versatile ancestors by mutation and gene loss associated with catabolic functions and acquisition of specialized virulence factors through mobile elements, as recently demonstrated for *Streptococcus equi* subsp. *equi* derived from the ancestor *Streptococcus equi* subsp. *zooepidemicus* [49]. Previously, Porcellato *et al*., based on differential analysis of virulence factors, highlighted that all *SDSE* from humans presented the genes coding for the C5a peptidase or the streptolysin O, while these genes were absent in horses, including those from this study. It appeared also that *SDSE* from horses had a horse-specific streptokinase, favouring tissue invasion [48].

In general, most of the isolates found in horses were from the genital tract (*n*=39/44). Frequently, *SDSE* are implicated in metritis in horses, causing infertility, but not always. For instance, isolates of the current study were found during a screening preceding *in vitro* fecundation, but mares did not develop metritis and were not treated. Such non-infective colonization could favour the silent dissemination of the HLGR mechanism. Gentamicin is the most widely used aminoglycoside in equine veterinary practices [50], and, in 2015, the European Medicines Agency provided harmonized criteria for its usage throughout Europe, limiting its application to respiratory infections affecting the lower respiratory tract caused by Gram-negative bacteria. However, at least in the past, gentamicin has been used for treating metritis [51], likely favouring the selection of HLGR*–SDSE* in the vaginal tract of horses. Besides, colonization of the genital tract by isolates colonizing the respiratory tract cannot be excluded. Dedicated investigations should be carried out to unveil the presence of HLGR determinants in pathogens or in commensals of the respiratory tract of horses.

## Conclusion

Characterization of the genetic background of HLGR in *SDSE* was unprecedented. This study unveils that genes related to HLGR are located on a mobilizable element, which presents a large host spectrum spanning from Gram-positive to Gram-negative species. This IME integrates into a preferred site represented by the *topA* gene of ICEs, predicting a considerable success of dissemination. HLGR–*SDSE* were able to disseminate among mares living in the same stud during 2 years. Dissemination of potential pathogens carrying these resistance determinants could represent a risk for horses’ health, considering that gentamicin is an important antibiotic to treat severe infections in equids. Although the *SDSE* lineage colonizing or infecting horses seems to have developed host specialization, in principle limiting its zoonotic potential, the transferability of HLGR from *SDSE* to human pathogens such as *S. agalactiae* by the transfer of the Tn*5252*-type ICE is a major concern for humans. HLGR is still uncommon but deserves to be monitored routinely, even in the frame of *in vitro* fecundation, to avoid latent dissemination.

## Supplementary material

10.1099/mgen.0.001722 Uncited Supplementary Material 1.

10.1099/mgen.0.001722 Uncited Supplementary Material 2.

## Abbreviations

BHI: brain heart infusion
CDSs: coding DNA sequences
GBS: group B *Streptococcus*
HLGR: high-level gentamicin resistance
ICE: integrative conjugative element
IME: integrative mobilizable element
nt: nucleotides
ONT: Oxford Nanopore Technologies
*SDSE*: *Streptococcus dysgalactiae* subsp. *equisimilis*
SLV: single locus variant
STs: sequence types
WGS: whole-genome sequencing
