# Supplementary material for: Integrative and conjugative elements carrying high-level gentamicin resistance genes in Streptococcus dysgalactiae subsp. equisimilis from horses
Source: Microb Genom. 2026 May 27;12(5):001722. doi: 10.1099/mgen.0.001722 (PMC13214566; doi:10.1099/mgen.0.001722)
Supplement: Uncited Supplementary Material 1. [file mgen-12-01722-s001.pdf]

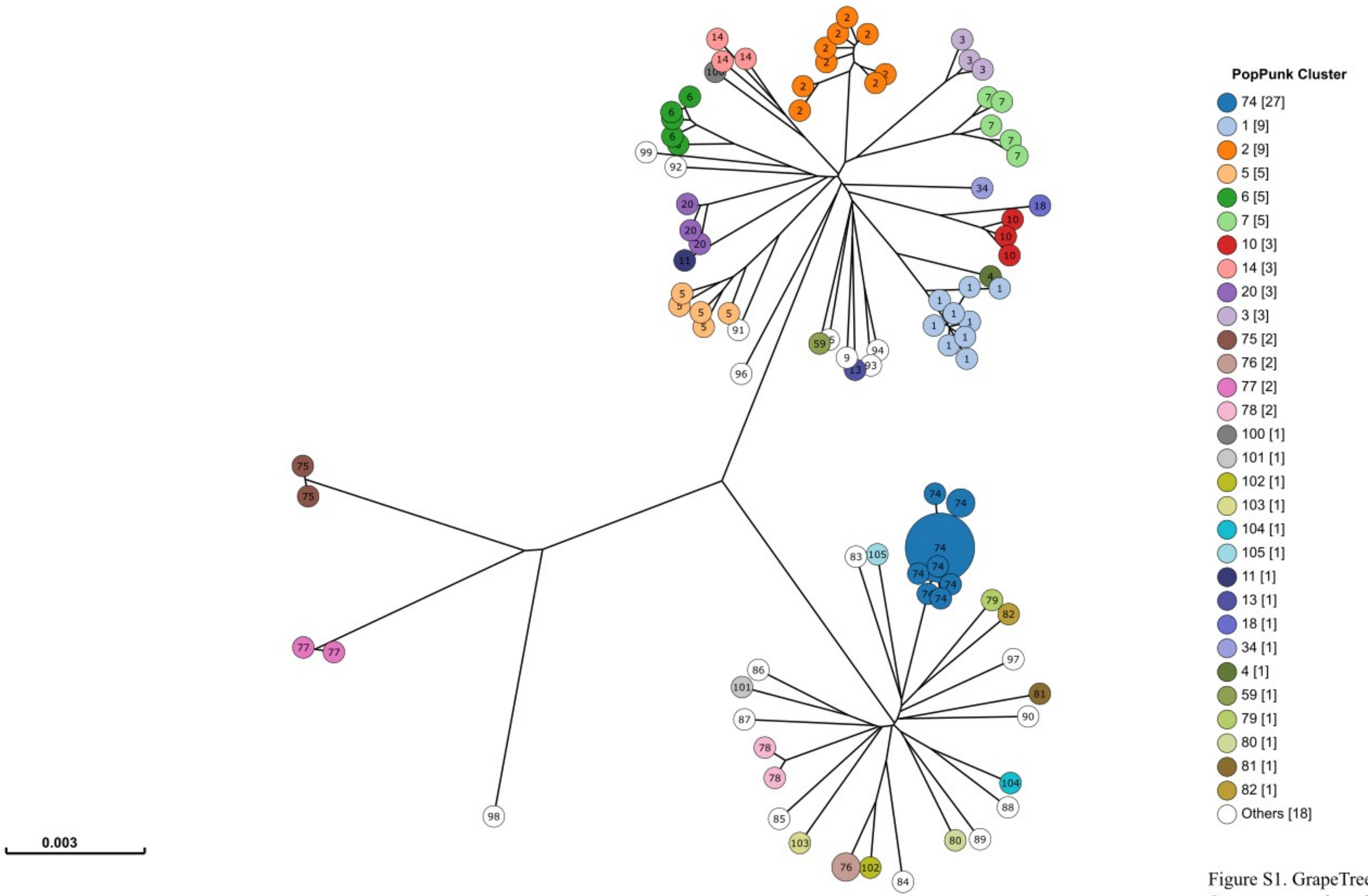

Figure S1. GrapeTree representation of PopPunk v2.7.8 cgMLST analysis of 114 *Streptococcus dysgalactiae* sbsp. *equisimilis* (*SDSE*). *SDSE* from French horses were grouped in cluster 74.

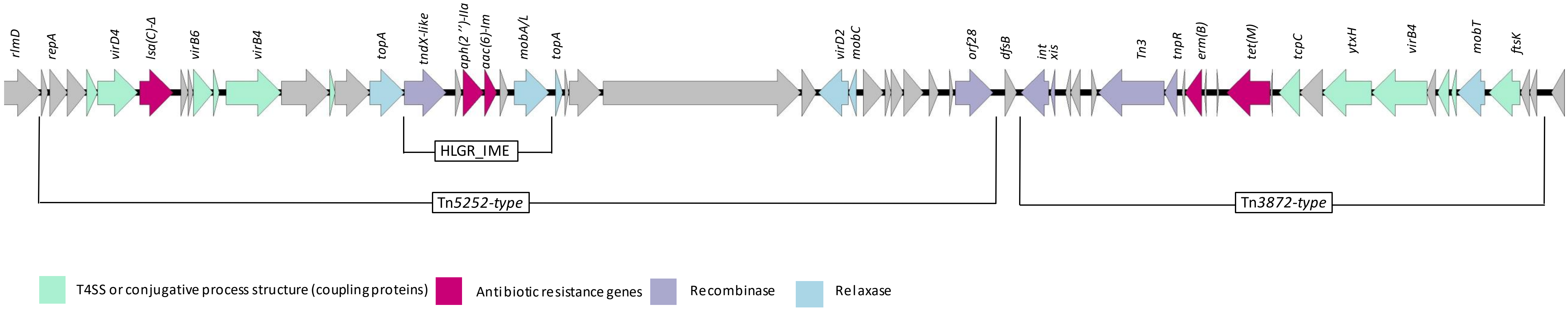

Figure S2. Rapresentation of Tn*5252*-type transfer in #63005 *Streptococcus agalactiae* (Easyfig v.2.2.5).
